# Decentralized P2P Electricity Trading Model for Thailand

**DOI:** 10.3390/s21217413

**Published:** 2021-11-08

**Authors:** Anchisa Pinyo, Athikom Bangviwat, Christoph Menke, Antonello Monti

**Affiliations:** 1 The Joint Graduate School of Energy and Environment, King Mongkut’s University of Technology Thonburi, Bangkok 10140, Thailand; anchisapinyo@gmail.com (A.P.); C.Menke@blv.hochschule-trier.de (C.M.); 2 Center of Excellence on Energy Technology and Environment (CEE), Ministry of Higher Education, Science, Research and Innovation (MHESI), Bangkok 10140, Thailand; 3 Department of Building Engineering Services, Trier University of Applied Sciences, 54293 Trier, Germany; 4 E. ON Energy Research Center, The Institute for Automation of Complex Power Systems, RWTH Aachen University, Mathieustr. 10, 52074 Aachen, Germany; amonti@eonerc.rwth-aachen.de

**Keywords:** decentralized electricity trading, local energy market, peer-to-peer, auction mechanism, Thailand energy transition

## Abstract

Thailand’s power system has been facing an energy transition due to the increasing amount of Renewable Energy (RE) integration, prosumers with self-consumption, and digitalization-based business models in a Local Energy Market (LEM). This paper introduces a decentralized business model and a possible trading platform for electricity trading in Thailand’s Micro-Grid to deal with the power system transformation. This approach is Hybrid P2P, a market structure in which sellers and buyers negotiate on energy exchanging by themselves called Fully P2P trading or through the algorithm on the market platform called Community-based trading. A combination of Auction Mechanism (AM), Bill Sharing (BS), and Traditional Mechanism (TM) is the decentralized price mechanism proposed for the Community-based trading. The approach is validated through a test case in which, during the daytime, the energy import and export of the community are significantly reduced when 75 consumers and 25 PV rooftop prosumers participate in this decentralized trading model. Furthermore, a comparison analysis confirms that the decentralized business model outperforms a centralized approach on community and individual levels.

## 1. Introduction

The power system transformation is a challenging global issue driven by 3Ds: Decarbonization, Decentralization, and Digitalization [1]. Decarbonization aims to reduce climate change impacts caused by carbon emissions. The power supply with a zero-CO_2_ strategy includes decarbonizing power resources and technologies, such as variable renewable resources (VRE), battery energy storage, and demand flexibility [2]. Decentralization introduces a more significant number of small power producers who produce and self-consume electricity generated by Distributed Energy Resources (DERs) called prosumers or prosumer communities. The consumption changes affect energy management and revenue loss of utility [3]. Finally, digitalization refers to the advancement of innovative technology and market solutions emerging in the power sector, as shown in the digitalization-based business models implemented in the European Union’s Horizon 2020 Research and Innovation program (EMPOWERh2020) [4]. 

These changes represent a significant impact on institutional, technical, and economic aspects of power systems. Thailand’s power system has been facing the transformation of the power system because of the following factors [5].

1. Outdated infrastructure and planning (powerplant and power line network).
2. Increasing number of Independent Power Supplies (IPS) who self-consume on their production sites.
3. Increasing RE integration (8476 MW in December 2015 to 10,949 MW in December 2018).
4. Increasing peak power demand (34,101 MW in 2018 to 34,317 MW in 2019).
5. Shifting of the peak demand from daytime to nighttime.

Hence, Thailand Power Development Plan (PDP) was revised on 30 April 2018 to include three main aspects: (1) Demand Response (DR) and Energy Management System (EMS); (2) renewable energy forecast; (3) Micro-Grid and Energy Storage System (ESS) [6]. The Alternative Energy Development Plan (AEDP 2018) was modified following the PDP 2018 to maintain 30% RE of total final energy consumption. The AEDP 2018 reduced the total RE contract capacity target from 19,634 MW by 2036 to 18,176 MW by 2037. The target of solar contract capacity was also adjusted from 6000 to 10,000 MW, including both ground-mounted and rooftop projects [6,7]. This represents the growing number of RE installations in the upcoming year, especially solar energy. 

Governmental organizations have continuously supported RE from solar by issuing incentive programs to encourage solar producers to join the program and sell their products through the distribution network. The first implementation was called “The adder scheme”, launched in 2007 for solar farms at 8 THB/kWh. The adder rate was reduced to be 6.5 THB/kWh in 2010. After that, the Feed-in-Tariff scheme was introduced in 2013 for both solar rooftops and solar farms. The incentive rates for solar rooftops are 6.16–6.96 in phase 1 and 5.66–6.85 in phase 2, while solar farms receive a fixed rate of 5.66 THB/kWh in phase 1 and 4.12 THB/kWh in phase 2. From 2013 to 2015, within this scheme, the PV generation was repurchased at a fixed higher rate than retail electricity price to facilitate PV installation [8]. In the middle of 2016, the Self-Consumption Pilot Scheme was announced for Rooftop PV. For this scheme, the PV generation must be consumed onsite, then any excess will be fed back to the main grid without any monetary credits [9]. On 4 October 2017, the self-consumption program was developed for repurchasing the excess PV rooftop generation to the main grid. The incentive rate for private households is 2.30–2.50 THB/kWh, while commercial, industrial, and large industrial stakeholders will receive 1.00 THB/kWh. From May 2019, Energy Regulatory Commission (ERC) launched the self-consumption program for residential PV rooftops to grant licenses at 1.68 Baht/kWh [10]. 

As mentioned above, the incentive programs have gradually reduced the buyback rate for subscribers. However, before participating in the incentive program, the subscribers will face limitations [11].

1. They have to turn a power meter into a bi-directional meter with THB 8500 as an additional cost.
2. The total amount of contract capacity in an area is limited due to the outdated trans-former (less than 15% of the nearby transformer size).
3. The overall contract capacity is limited, and the incentive price is reduced due to the impact of repurchasing pass-through energy costs on customers’ electric bills [9,12].
4. The repurchasing time of the current scheme is too short (10 years) comparing to the previous scheme (20–25 years).
5. They have to notify the third party for modifying their home or building.

However, some small prosumers who would like to sell the PV production need to participate in the incentive program despite many limitations. This case happened because Thailand’s power market has been under the Enhanced Single Buyer model [13], so the small producers cannot sell the energy production to others except the authorized utility company. Moreover, they cannot sell surplus power production over the power capacity specified in the Power Purchasing Agreement (PPA). Therefore, they will lose the excess production without compensating if their PV rooftop systems produce power over the contract capacity. As a result, many rooftop owners decide to install on-grid PV systems, but they are not interested in joining the government sector’s incentive program. Therefore, this paper aims to introduce a decentralized business model to lead a decentralized energy trading system in Thailand’s power market. However, there have been a number of studies on P2P business models, which include different bidding and auction schemes. The models are proposed and simulated separately. Long, C., et al. [14] proposed three different market paradigms for a P2P energy trading. Those were Bill Sharing (BS), Mid-Market Rate (MMR), and Auction-based Pricing Strategy (APS). The clearing prices were determined differently for three scenarios. The clearing price was set to the lowest selling price offered by the sellers in the case that PV generation is more than demand. The highest offer price is the clearing price, when PV generation equals demand. For the case that PV generation exceeds demand, the highest bid price is assumed as the clearing price. The proposed mechanisms were evaluated as they were implemented separately. A peer-to-peer (P2P) energy trading system among prosumers is introduced by Hien Thanh Doan, et al. [15], using a double auction-based game theoretic approach to improve prosumers’ profits and reduce impacts on the grid. The approach was simulated under different scenarios to demonstrate the effectiveness of the method. A platform for P2P energy trading in a micro-grid, called “ElecBay”, based on an eBay-style business model, was proposed by Zhang C., et al. [16]. The model was simulated to investigate the behavior of energy prosumers. It was found that the model was able to balance local generation and demand, and to facilitate a large penetration of renewable energy resources in the grid.

This paper introduced a combination of electricity trading mechanisms for P2P circumstances in Thailand, which can be summarized as follows:

1. The combination of trading schemes is designed to encourage P2P power exchange and cope with the low buyback rate. The price mechanism is the combination of three mechanisms: Auction Mechanism (AM), Bill Sharing (BS), and Traditional Mechanism (TM).
2. As the buyback rate is rather low, the proposed trading scheme enables maximum benefits for sellers, whereas the electricity can be sold at a higher price than the buyback rate. Simultaneously, the buyers have a chance to buy electricity at a lower price than the grid price.
3. This model increases the independence of prosumers and consumers in Thailand’s power market. The auction method is designed to allow maximum trading quantity at clearing price, which satisfies both buyers and sellers. The unmatched quotations from buyers and sellers are then processed in the sharing mechanism. Consequently, the remains from the first two mechanisms are traded with the grid as the TM method.

In Section 2, the authors present the system design. Then, assessment parameters are explained in Section 3, while simulation and results are discussed in Section 4. Finally, the conclusions and future work are summarized in Section 5.

## 2. System Design

This section will detail system architecture, process scheduling, and trading algorithms in the decentralized electricity trading model. The system architecture is designed based on the Local Energy Market (LEM) [16,17,18,19,20,21], a marketplace to trade local electricity from peers with surplus energy to deficit peers within a community. LEM has different three structures based on market interactions [16]. Firstly, Fully P2P, a market structure where sellers and buyers directly trade energy without any intermediary. Secondly, Community-based, a market structure where sellers and buyers exchange energy on a market platform. The market platform is a local energy marketplace controlled by an intermediary who plays a crucial role in energy allocation and price-making. Finally, Hybrid P2P is a market structure where sellers and buyers can decide to exchange energy on the market platform or with each other. The suitable market structure for this introduced model is Hybrid P2P because the model allows prosumers and consumers to select trading options and trading partners independently. They can choose to whom they would like to exchange energy, for example, nearby neighbors, electricity market platform, or utility grid. Moreover, they can set trading quantity and electricity price by themselves to represent a free-market structure for Thailand’s power market. Therefore, the next step will detail system architecture to describe the market components and interactions in the decentralized electricity trading model. 

### 2.1. System Architecture

The system architecture [17] consists of physical components such as players, Distributed Energy Resources (DERs), power meters, distribution networks, and connection points on the physical layer. This system design has two different groups of players—users and platformers. A user is a homeowner who would like to participate in the decentralized electricity trading system as either a seller or buyer. This system assumes that the small producers install solar PV systems on their rooftops to produce electricity for sale and self-consuming. Each small producer must consume electricity produced onsite before selling the excess production to others, also called a prosumer. Therefore, a prosumer with surplus energy for sale plays a role as a seller, while the buyer is a consumer or prosumer with insufficient power. Another group of players is a platformer, an intermediary with an essential role in energy allocation, price-making, and providing ancillary services on the electricity market platform. The platformers can be Distribution System Operators (DSOs), Balance Responsible Parties (BRPs), Aggregators, and retailers [21,22]. This model presents them with a choice to create a new business in the power sector. This system does not have other energy resources except solar rooftops and utility grids, so the PV homes must connect to the others to balance energy in their homes. The whole system must also connect to the outside utility grid to balance energy inside the trading area. Power flows transfer through the existing physical network where power transfers are allowed in two directions—import and export. The connection points install bi-directional power meters to measure import and export energies at individual prosumer’s homes and the Point of Common Coupling (PCC). Consumers do not need bi-directional power meters because they need only one direction for the imported energy.

Another layer in the system architecture, the information layer, consists of virtual components that enable the physical elements to interact and operate the data submitted from the physical parts. Users and power meters submit data to the information layer, including price mechanisms to create market interactions and operate the data submitted from the physical elements. This paper assumes that the energy marketplace is an online platform where users can meet trading partners and trade energy online. First, the users need to connect their hardware devices to the energy market platform, for example, mobile phones, laptops, or personal computers. Next, each user submits an energy order via the hardware device that will then process the order on the energy market platform in which users can monitor their trading status online. This paper focuses on the trading algorithm, which is a vital part of the energy trading platform. Therefore, the following section will schedule the trading process in sequences before detailing the algorithm.

### 2.2. Process Scheduling

**Figure 1:**
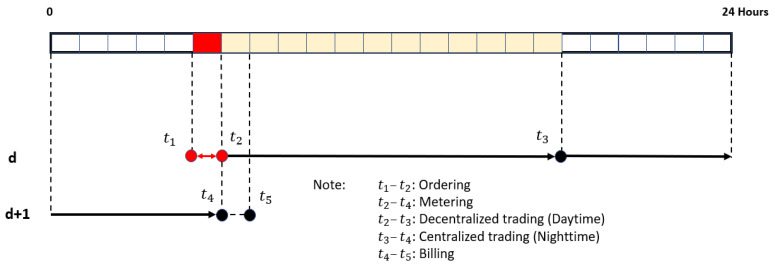
Process scheduling.

The decentralized electricity trading model includes three main steps of the trading process running in sequences from the beginning to the end: 1. Ordering, 2. Metering, and 3. Billing [16]. Users need to understand a basic trading process in the energy market platform to participate practically. Figure 1 demonstrates that each step of the trading process is scheduled with a particular time interval each day. The first step, Ordering, takes one hour, starting from market opening time ($t_{1}$) to market closing time ($t_{2}$). In this step, users create and submit their energy orders to the energy market platform before market closing. The market platform will then collect the energy orders for calculation later. The second step, Metering, continuously runs when the first step ends, takes 24 h from the market closing time today ($t_{2}$) to tomorrow ($t_{2}$).

**Figure 2:**
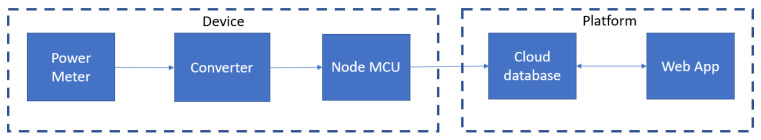
A power meter connects to the market platform.

In this step, power meters installed at every connection point measure import and export energies and then submit them to the energy market platform. Each power meter connects to the market platform following the diagram shown in Figure 2. The meter connects to a converter to convert signals to communicate with a NodeMCU, a controller board that can communicate with other devices by using Wireless LAN (WLAN). In this paper, the NodeMCU is programmed to take a reading from the power meter and push the reading data to a cloud database every 12 h (at 6 AM and 6 PM). Then, the webserver reads recorded data on the cloud database and displays it on the web browser. The metering data from a power meter is accumulated data, so the web server must add a function to compute net energy used every 12 h. This paper divides the Metering into two sub-periods, Daytime (12 h) and Nighttime (12 h), because this paper considers only energy supply by PV rooftop systems. The PV rooftop prosumers can provide available energy for sale in the energy market platform during the daytime. However, they must consume power from the utility grid during the nighttime because their PV system cannot produce electricity. Therefore, with the PV systems, the users can participate in decentralized electricity trading only during the daytime. At night, they must join in centralized trading. After completing the Metering step at $t_{4}$, the energy market platform takes the required data collected from the previous processes, then computes them by a trading algorithm implemented on the market platform. The final step, Billing, takes one hour, starts from $t_{4}\;\mathrm{to}\;t_{5}$. In this step, the market platform collects results computed by the trading algorithm, then calculates daily trading reports for individual users. The following step will detail the trading algorithm, the most important module to enable decentralized electricity trading among end-users in an energy trading system.

### 2.3. Trading Algorithm

As mentioned previously, this system considers only PV rooftop systems installed in the individual sites of small prosumers who are one type of market player. Therefore, this introduced trading algorithm computes submitted data from physical components in different methods; decentralized trading is a method for data submitted during the daytime, and centralized trading is a method for data submitted during the nighttime. Furthermore, the decentralized trading method applies the Hybrid P2P market to enable market players to trade their electricity on the energy market platform with and without a price-maker. It consists of two trading options: Fully-P2P and Community-based trading. The trading algorithm implemented on the energy market platform was designed based on three steps of the trading process: Ordering, Metering, and Billing. The data inputs from the physical layer are submitted to compute in the trading algorithm, consisting of the energy orders from users and the metering data from power meters. 

**Figure 3:**
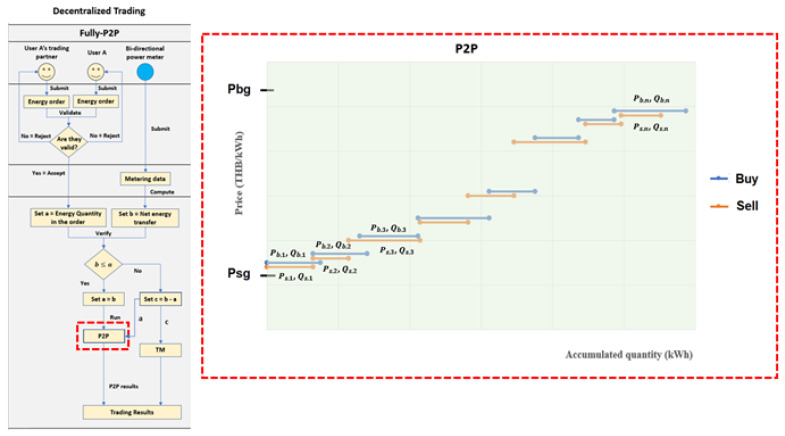
Fully-P2P trading.

**Figure 4:**
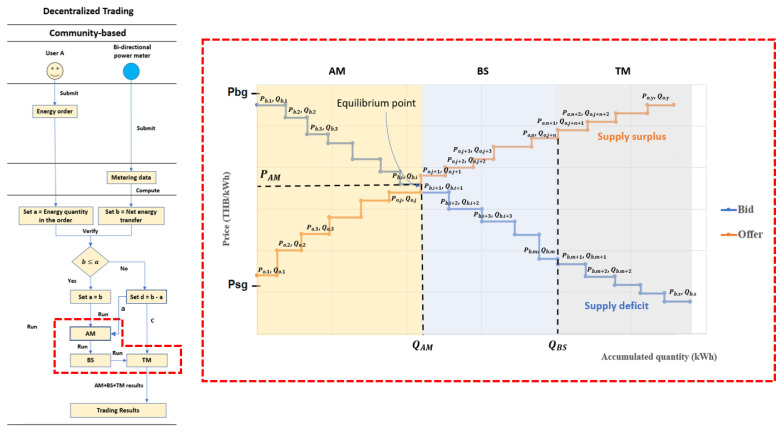
Community-based trading.

The energy market platform runs the decentralized trading method from Ordering to Billing steps during the daytime. The Ordering starts at 5 a.m., allows users to submit energy orders to the market platform by 6 a.m. Before submitting the energy orders, users decide which trading options they would like to join—Fully-P2P or Community-based. If they choose the first option (Figure 3), they must first negotiate with their trading partners and summarize the negotiating results as energy orders. Then, each pair of traders and partners must report the energy orders to the energy market platform. Next, the market platform validates the energy orders. Each pair of energy orders needs consistency in energy price, quantity, and trader names (seller and buyer). The market platform will then accept only valid energy orders for the following process. Otherwise, the market platform will reject them and notify the owners of the error indicated in the energy orders. If the users choose the second option (Figure 4), they can create an energy order for themselves without any matching and negotiation beforehand. Then, the market platform accepts the submitted orders and collects them for calculation in the following process. After 6 a.m., the energy market platform closes the order submission and starts the Metering step. With decentralized trading, the energy market platform considers metering data submitted from power meters from 6 a.m. to 6 p.m. First, each power meter measures import and export energies through its connection point, then sends them to the market platform in the form of accumulated values at the metering time. After that, the market platform collects the metering data and computes net energy consumed or sold by individual households and the whole system from 6 a.m. to 6 p.m. The net energy determines its role in energy trading (seller or buyer) and represents actual energy transferred for individual households. The next step is pairing the net energy transfer with the energy order owned by the same user. Then, the energy market platform compares the net energy transfer (b) with the energy quantity (a) specified on the corresponding energy order. The market platform needs values a and b to compute the algorithms.

Figure 3 illustrates the first trading option (Fully-P2P trading). If the value b does not exceed value a, the market platform will replace a by b and compute the modified energy order by the first price mechanism, called Peer-to-Peer (P2P). On the other hand, if the value b exceeds value a, the market platform will compute the excess value (c) in Traditional Mechanism (TM). The P2P, a price mechanism with the highest decentralization degree, is implemented in the Fully-P2P trading option. After modifying the energy orders by using the actual energy transfer, a pair of energy orders are the same price ($P_{b,1}$ = $P_{s,1}$, $P_{b,2}$ = $P_{s,2}$, $P_{b,3}$ = $P_{s,3}$, …, $P_{b,n}$ = $P_{s,n}$), but their quantities do not need equality because we apply the actual transfer. The clearing prices within the P2P price mechanism are the quoted price for each pair order and range between retail price (Pbg) and buyback price (Psg), as demonstrated in Figure 3. Therefore, users who participate in the Fully-P2P option obtain the exact price they satisfy.

**Figure 5:**
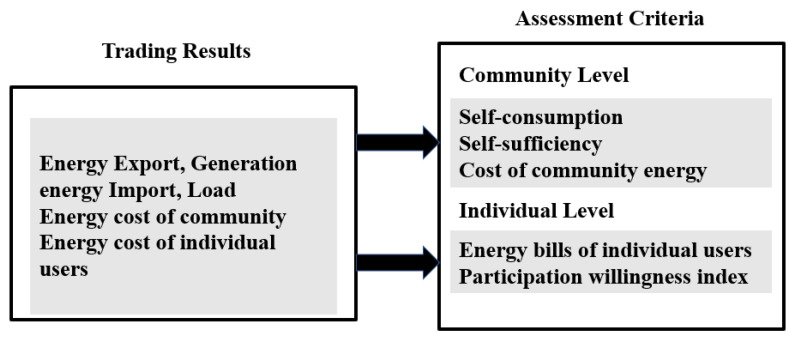
Two levels of model assessment.

Figure 4 illustrates the second trading option (Community-based trading), if the value b does not exceed value a, the market platform will replace a by b, then combine the modified energy order with the excess orders from the first option. After that, the market platform computes all orders by the second price mechanism, called AM + BS + TM [23]. On the other hand, if the value b exceeds value a, the market platform will calculate the excess value (d). The surplus-value of both demand and supply sides will then trade by centralized price mechanism or Traditional Mechanism (TM). Auction Mechanism + Bill Sharing + Traditional Mechanism (AM + BS + TM), a price m14echanism with a lower decentralization degree than the P2P, is implemented in the Community-based option on the energy market platform. The AM + BS + TM algorithm combines three computational methods for running data inputs in sequence, as demonstrated in Figure 4. Each computational method allocates energy quantity and computes clearing price separately. The first step, energy orders submitted by users who participate in the Community-based option and the excess orders from the first option are set as data inputs, consists of bids and offers. Then, the market platform sorts bids and offers based on their prices [20]. The bids rank in descending prices ($P_{b,1}>P_{b,2}>P_{b,3}\;\ldots >P_{b,x}$). The offers rank in ascending prices ($P_{o,1}>P_{o,2}>P_{o,3}\;\ldots >P_{o,y}$). After that, the Auction Mechanism runs the sorted inputs and computes the clearing price and quantity at the equilibrium point, where all offer prices do not exceed the bid prices and the amount of accumulated energy in bids equals the amount of accumulated energy in offers ($Q_{b,1}+Q_{b,2}+Q_{b,3}+\ldots +Q_{b,i}=Q_{o,1}+Q_{o,2}+Q_{o,3}+\ldots +Q_{o,j})$ [14,17,24,25,26]. In this paper, the clearing price for the Auction Mechanism is set at a bid price on the equilibrium point ($P_{AM}=P_{b,i}$), while clearing quantity ($Q_{AM}$) shows the maximum executable volume to balance demand and supply on the market with considering electricity prices. The energy orders on the left of the equilibrium point show executable orders by Auction Mechanism. The owners who own all of these energy orders are satisfied to trade electricity at the clearing price because they can consume electricity at a lower price or sell electricity at a higher price than expected. The Auction Mechanism cannot execute the energy orders on the right of the equilibrium point, so the Bill Sharing Mechanism will further run them. The excess bids and offers still rank based on their prices, but bid prices are lower than offer prices in this zone. The Bill Sharing computes clearing price, and quantity within energy balancing zone where the amount of total energy supply equals the amount of total energy demand without considering electricity prices ($Q_{b,1}+Q_{b,2}+Q_{b,3}+\ldots +Q_{b,m}=Q_{o,1}+Q_{o,2}+Q_{o,3}+\ldots +Q_{o,n})$ [14,27,28]. The energy orders in the blue area, Figure 5, show executable orders by Bill Sharing Mechanism. With Bill Sharing, the clearing price ($P_{BS}$) is a weighted average of offer prices from the executable offers. Therefore, the equation can be derived as follows:

(1) $$P_{BS}=\frac{P_{o,j+1}Q_{o,j+1}+P_{o,j+2}Q_{o,j+2}+P_{o,j+3}Q_{o,j+3}+\ldots +P_{o,j+n}Q_{o,j+n}}{Q_{o,j+1}+Q_{o,j+2}+Q_{o,j+3}+\ldots +Q_{o,j+n}}$$

In Bill Sharing, the sellers who own offer prices lower than the BS’s clearing price ($P_{BS}$) are satisfied to trade at this point because they can get more income than they expected. For example, if $P_{BS}\;\mathrm{is}\;P_{o,j+3},$ the sellers who own $P_{o,j+1}$ and $P_{o,j+2}$ are satisfied to trade at $P_{o,j+3}$ because $P_{o,j+3}$ is higher than their offer prices.

On the other hand, the sellers who own offer prices higher than the BS’s clearing price ($P_{BS}$) and all buyers who own energy orders traded in the Bill Sharing are unsatisfied with trade because it does not meet their expectations. For example, if $P_{BS}\;\mathrm{is}\;P_{o,j+3},$ the sellers who own $P_{o,j+4}$, …, $P_{o,j+n}$ are unsatisfied to trade at $P_{o,j+3}$ because $P_{o,j+3}$ is lower than their offer prices. Likewise, the buyers who own $P_{b,i+1}$, $P_{b,i+2}$, …, $P_{b,i+m}$ are unsatisfied to trade at $P_{o,j+3}$ because $P_{o,j+3}$ is higher than their offer prices. 

However, they can accept the results because the BS’s clearing price ($P_{BS}$) is better than buyback and retail prices in Traditional Mechanism (TM) [9]. As a result, the energy orders on the right of the energy balancing point represent supply surplus or supply deficit, which cannot operate in the Bill Sharing. After that, the market platform computes the excess orders from both cases by Traditional Mechanism (TM) to balance energy demand and supply inside the trading system. In Traditional Mechanism, the excess offers ($Q_{o,n+1},\;Q_{o,n+2},\;\ldots Q_{o,y}$) are sold at a fixed buyback price ($P_{sg}$), and the extra bids ($Q_{b,m+1},\;Q_{b,m+2},\;\ldots Q_{b,x}$) are bought at a fixed retail price ($P_{bg}$).

Additionally, on the right, is a centralized price mechanism or Traditional Mechanism (TM) implemented in the centralized trading method during the nighttime. In this trading method, the energy platform does not need the submission of energy orders from users. Instead, the market platform needs only metering data from the power meters at every connection point to compute net energy transfer for individual households and the whole system from 6 p.m. to 6 a.m. The net energy transfer from all users presents only the demand side during the nighttime because PV producers cannot generate enough electricity for sale inside the trading system. After that, the energy market platform will then compute the net energy transfer by the centralized price mechanism or Traditional Mechanism (TM).

After successfully running both decentralized and centralized trading methods, the market platform enters the final step, Billing. In this step, the market platform collects trading results from these price mechanisms, computes daily trading reports, and sends them back to individual users by 7 a.m. every day. The users and platformer can check total costs, income, and trading performances, so they can adjust the input data the next day to improve trading performance. In addition, the trading reports include service charges and taxes. The platformer collects the service charge as an income, while the tax is collected from buyers’ costs for a relevant organization outside this trading system. Finally, the market platform records the trading reports and computes monthly bills for individual users. 

## 3. Model Assessment

The model assessment for this paper is categorized into two primary levels [27,29], as shown in Figure 5. There are three community level criteria: the self-consumption ratio, self-sufficiency ratio, and energy costs. The individual energy bill and the P2P participating willingness index are set as assessment criteria on the user level. The decentralized electricity trading model was evaluated compared to the centralized model set as a base case. 

### 3.1. Self-Consumption

The community’s self-consumption refers to how much PV production is used to serve the Micro-Grid’s demand [28]. The self-consumption under centralized model ($SC_{Cen}$) is calculated by the ratio between the summation of individual self-consumption consumed by prosumers ($n_{p}$) and the overall PV production during the particular time, while the self-consumption under decentralized model ($SC_{De}$) is calculated by the ratio between the summation of individual self-consumption consumed by the community and the overall PV generation produced by the community. The formulae for the calculation are described in Equations (A1)–(A3), Appendix A. Both values of $SC_{Cen}$ and $SC_{De}$ rank between 0 and 1. The higher value implies how significant the self-consumption is. 

### 3.2. Self-Sufficiency

The community’s self-sufficiency refers to how much the whole electric load can be supplied by total PV production in the community [27,29].

Under both centralized and decentralized schemes, the self-sufficiencies ($SS_{Cen}\;\mathrm{and}\;SS_{De}$) are calculated by the ratio between the amount of total electric loads that are supplied by the overall PV generation produced by the community and the amount of total electric loads in the community during the particular time. The formulae for the calculation are described in Equations (A4)–(A6), Appendix A. Both values of $SS_{Cen}$ and $SS_{De}$ rank between 0 and 1, which indicate no self-sufficiency to the full self-sufficiency.

### 3.3. Cost of Community Energy

Only prosumers can participate in a centralized model, reducing electric cost by self-consumption and gaining income by selling the excess supply to the bulk utility grid at a fixed buyback rate ($P_{sg}$). All consumers still buy energy to meet their demand at the retail rate ($P_{bg}$) as usual. In a decentralized model, both prosumers and consumers can participate as traders on the energy market platform, so consumers can consume the excess supply from prosumers in the community before buying the residual demand from the bulk utility grid at a fixed retail price rate ($P_{bg}$). Likewise, prosumers also sell the excess supply after trading in the community to the main grid at a fixed buyback rate ($P_{sg}$). Thus, community energy cost under a decentralized model is less than the community’s cost under a centralized model. The formulae for the calculation are described in Equations (A7) and (A8), Appendix A.

### 3.4. Energy Bills of Individual Users

Both energy import and export possibly happen for prosumers each day, while the energy import happens only for consumers each day. Moreover, a user who participates in a decentralized model commonly spends the energy bill less than a user who participates in a centralized model because of the better electricity price and the self-consumption of energy. The formulae for the calculation are described in Equations (A9) and (A10), Appendix A.

### 3.5. Participation Willingness Index

The participation willingness index is defined as the percentage of users who pay less for energy after participating in the decentralized model [27,29]. The formulae for the calculation are described in Equation (A11), Appendix A. This index shows an ability to keep the number of participants who trade electricity under a decentralized model.

## 4. Simulation and Results

This section will detail the simulation approach, testing approach, and results. This paper assumes that 100 units of residential households are set as a Micro-Grid for testing the decentralized electricity trading model as described in Section 2. The assumed community micro-grid is located in a metropolitan area, Thailand. The 100-household system consists of 25 prosumers who generate electricity from their solar rooftop systems and 75 consumers who consume electricity from supply sources—prosumers or the utility grid. 

### 4.1. Simulation Approach

**Figure 6:**
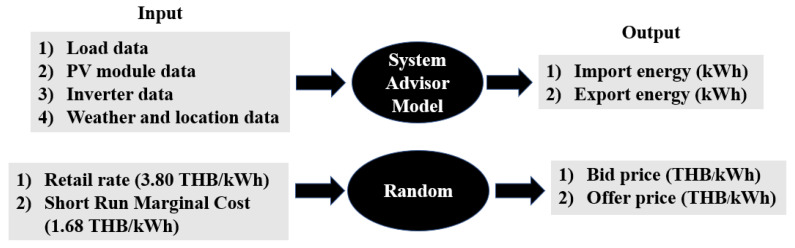
Creating sample data.

The sample data is created following the procedure as demonstrated in Figure 6. The sample data are import and export energies of the assumed 100 households and energy prices. The import and export energies are created by using the System Advisor Model (SAM) 2020.11.29 Revision 1, SSC 252 [30], which requires four inputs: the hourly load data, PV module data, inverter data, and weather and location data. The hourly load data for 100 households are generated based on the consumption of residential customers in MEA’s area (less than 150 kWh per month and more than 150 kWh per month) in 2019 [31,32]. The 25 prosumers install PV systems of 3.352 kWdc capacity, with 10 monocrystalline PV modules (Sun Power SPR-X21-335) and an inverter (SMA SB 3000TLST-21 230 V, 1 ph, 50 Hz, AC to DC ratio of 1.32). Thus, the total AC capacity is 2.540 kW. PV modules’ annual DC degradation rate is 0.5% of the total annual energy output (kWh). In addition, the European Commission Photovoltaic Geographical Information System (PVGIS) provides weather and location data that covers Europe, Africa, Central Asia, and parts of Southeast Asia [33]. 

Another required data set is energy prices, bid price, and offer price (THB/kWh). In general, bid and offer prices should be generated based on energy production costs and demand–supply forecasting data as a role of the energy management system. Users need to create better prices to get better trading results. However, this paper focuses on the decentralized electricity trading algorithm deployed on the market platform, so the algorithm for generating bids and offer prices is out of scope. Thus, bid and offer prices are random numbers between [1.68, 3.80], which is the buyback price ($P_{sg}$) and retail electricity price ($P_{bg}$), respectively. The retail electricity price ($P_{bg}$) represents the average residential electricity costs per unit during 2019 in the metropolitan area [34]. The buyback price ($P_{sg}$) represents a Short Run Marginal Cost (SRMC) [12]. The simulated energies are scheduled based on trading periods, decentralized trading in the daytime, and centralized trading at nighttime. This paper sets 6 a.m. to 6 p.m. for decentralized trading and 6 p.m. to 6 a.m. for centralized trading. The simulated energies and energy prices will be computed in the trading algorithm as described in Section 2.3. Two pairs of households (HH 24–HH 27) are set to participate in the Fully-P2P option. The other 94 families (HH 1–HH 23, and HH 28–HH 100) are selected to participate in the Community-based option. The trading results will be detailed in the following section.

### 4.2. Energy Allocation and Price Calculation in Decentralized Electricity Trading Model

**Figure 7:**
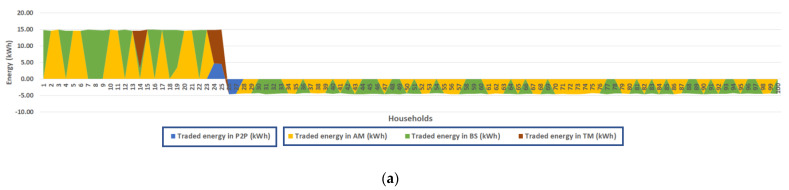

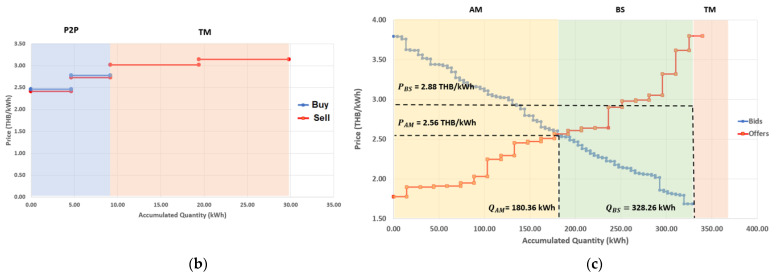
(**a**) Energy allocation in decentralized trading model. (**b**) Fully-P2P trading option. (**c**) Community-based trading option.

The energy market platform implements the decentralized electricity trading model to enable participants to exchange energy supplies and demands to nearby neighbors in an energy sharing system (Micro-Grid) or utility grid independently. Within this model, a Hybrid-P2P, participants can select the trading alternatives they are willing to participate in, for example, Fully-P2P or Community-based trading options. Figure 7a demonstrates total energy traded inside the simulated trading system from 6 a.m. to 6 p.m. on 9 April. Total energy supply after self-consuming provided by 25 prosumers is 369.50 kWh, while total energy demand from 75 consumers is 337.45 kWh. Therefore, the energy market status represents supply surplus on this day. Different colors in the area chart illustrate the energy allocation according to the implemented trading options. For example, two pairs of participants choose the Fully-P2P option to exchange electricity with each other, a pair is HH24 and HH26, and another pair is HH25 and HH27. HH24 and HH25 agree to sell PV production to meet energy demand from HH26 and HH27, as shown in the blue area in Figure 7a. 

Then, they report the negotiation results to the market platform in the form of energy orders. They submit the same energy price and quantity as their trading partners. After that, the orders are modified by actual transferred energy from power meters. Thus, the quantities in the pair orders may not be the same. However, this paper assumes that the submitted quantities are the same as transferred energy from power meters. Figure 7b demonstrates that HH24 exchange energy to HH26 at a price of 2.46 THB/kWh, and HH25 exchange energy to HH27 at a price of 2.78 THB/kWh. Both pairs agree on the trading prices which range between buyback and retail rate. Thus, users who participate in Full-P2P trading get the better price than centralized trading. As shown Figure 7a,b, HH24 and HH25 still have excess energy after trading in the Fully-P2P option. Therefore, the surplus is sold to the utility grid at a fixed buyback rate (1.68 THB/kWh) called Traditional Mechanism (TM). 

Figure 7a also demonstrates available energy supply and demand from other households who select the Community-based option. The market platform gathers the energy orders (Bids and offers) from households and runs them through the AM + BS + TM price mechanism in sequence, as shown in Figure 7c. The different colors in Figure 7c relate to the colors on the area chart in Figure 7a to identify the trading methods for each household. 

In the first step, AM computes clearing price and quantity at the equilibrium point where all offer prices are less than bid prices. The example case shows the clearing price is 2.56 THB/kWh, and the clearing quantity is 180.36 kWh. This result demonstrates that all participants who trade under the AM method (in the yellow area) exchange their supply and demand at a clearing price for each Auction round. At the AM clearing prices, they can receive better benefits than their expectations. After that, the BS method will then compute the excess energy orders behind the equilibrium point. Figure 7c illustrates the BS’s trading results in the green area in which total energy supply equals demand, 328.26 kWh in this case. Therefore, this area represents total energy orders that can trade under the BS method. 

The BS’s clearing price is the weighted average of offer prices located in this green area, for example, 2.88 THB/kWh in this case. The BS’s clearing price is logically higher than AM’s clearing price because of the higher offer prices in the green area. Thus, the sellers who submit offer prices less than the BS’s clearing price are satisfied because they get income greater than their expectation.

On the other hand, the sellers who own the higher prices and all buyers in the green area are unsatisfied because they will benefit less than their expectations. However, they can accept the BS’s results because it is better than participation in the TM method, a centralized trading approach. Finally, the TM method computes the excess energy after trading under the BS. For example, in this case, the energy market will sell surplus supplies to the utility grid at a fixed buyback rate, 1.68 THB/kWh. Finally, the TM method computes the excess energy after trading under the BS. For example, in this case, the energy market will sell the excess supplies to the utility grid at a fixed buyback rate, 1.68 THB/kWh. Another possibility, in supply deficit, the energy market will let them buy electricity from the utility grid at a fixed retail rate, 3.80 THB/kWh.

### 4.3. Comparison between Decentralized and Centralized Electricity Trading Model 

This section will detail the differentiation after applying decentralized and centralized electricity trading models in the simulated Micro-Grid. First, the centralized model is set as a base case and compared using five assessment criteria described in Section 3. The first three parameters evaluate at the community level, and the following two parameters test at the individual level. 

The first parameter is self-consumption, representing the energy used to serve the community Micro-Grid’s total demand. In the decentralized trading model, prosumers can consume energy production themselves before selling the excess energy to consumers in the community. In contrast, prosumers who participate in the centralized trading model cannot sell the extra power to others in the community. Therefore, the decentralized trading model shows a higher self-consumption rate than the centralized model at the community level significantly. The second parameter is Self-Sufficiency, which refers to the energy consumption from electric loads that the energy production in a community Micro-Grid can supply. 

In the centralized trading model, players cannot consume energy production from other players in the Micro-Grid except themselves. Therefore, they need energy outside to reduce the scarcity. On the other hand, the decentralized trading model allows players to consume energy inside the Micro-Grid before trading outside. The results show that the decentralized model gets a higher score of self-sufficiency rate than the centralized model because the higher amount of the community’s demands can be covered by the PV production. Both parameters look similar, but they have slightly different meanings. For example, the first parameter considers the energy production consumed by the community, but the second parameter considers the load consumption handled by the community’s energy. The third parameter is the community’s energy costs, which weighs up only events where the community consumes energy outside. The numbers in Table 1 indicate the energy cost of the community from 6 a.m. to 6 p.m. in year one. The results show that the decentralized trading model’s community pays less energy cost than the community with the centralized model implementation because the decentralized model allows participants to consume energy from the community’s members before trading outside. As a result, the community self-consumes more energy inside, so it consumes minor energy outside.

At an individual level, the fourth parameter is the average cost of individual users, which refers to the cost of energy consumed by individual users when they participate in the different trading models. In Table 1, the positive sign demonstrates energy cost, but the negative sign shows energy income. The results show that consumers and prosumers who participate in the decentralized trading model get more benefits than participation in the centralized model. Consumers get slightly different costs, but prosumers earn heavily different incomes because the energy selling price increases within the decentralized model. The last parameter is the participation willingness index, which indicates the proportion of the satisfied participants to exchange energy in the decentralized trading model. The centralized trading model is set as a base case to compare with the decentralized trading model, so the participation willingness index of the centralized model is zero. The result shows that all users are willing to participate in the decentralized model because they reduce energy costs and earn much more income than participation in the centralized trading model. 

## 5. Conclusions and Future Work

It has been customary that electricity customers buy their power from the grid, the so-called Traditional Mechanism (TM) in this paper, at a fixed retail rate of 3.80 THB/kWh. The electricity produced by a residential PV rooftop system in Thailand is mainly intended for self-consumption. The grid will buy the excess from self-consumption with a buyback rate of 1.68 THB/kWh. Direct exchange of electricity between households is not permitted. Hence, the decentralized business model was proposed for trading electricity in a Local Energy Market.

Households can exchange electricity in the community Micro-Grid through the market platform deployed by a decentralized market mechanism, divided into three steps—Bidding, Metering, and Billing. Three pricing mechanisms are established to accommodate bids and offers of households: Auction Mechanism (AM), Bill Sharing (BS), and Traditional Mechanism (TM).

The AM allows priority to the higher bids and lower offers until the equilibrium price is reached. The bids for lower prices and the offers for higher prices than the equilibrium price will not be traded in this mechanism but are postponed to the bill sharing mechanism. The BS method maximizes the trading volume. If the total amount of bids equals the total amount of offers, the clearing price will be the weighted average price of the offers. In case of surplus and deficit, the unmatched offers will be sold to the grid at the buyback rate of 1.68 THB/kWh, and the unmatched bids will be drawn from the grid at 3.80 THB/kWh.

A set of parameters was developed to assess the decentralized system’s performance and compare it with the traditional centralized model. On the community level, self-consumption, self-sufficiency, and cost of community energy were considered. For user level, energy bills of individual users and participation willingness index were assessed.

An estate of 100 residential units with a micro-grid system was simulated. They consisted of 75 consumers and 25 prosumers, and each was equipped with 3.325 kWdc solar PV rooftop systems. At the community level, the proposed model gets the significant self-consumption rate, self-sufficiency rate, and cost reduction to the community Micro-Grid. Moreover, a participant who joins the decentralized model as a prosumer can get significantly high benefits. A consumer can reduce the individual energy bill with the decentralized model and receives a 100% participation willingness index on both demand and supply sides. In conclusion, the decentralized business model benefits both community and individual levels more than the centralized business model.

Based on the work in this paper, the following directions are identified for further study:

(1) According to Table 1, the decentralized trading model significantly reduces energy import and export of community, but other factors are reflecting the amount of energy import and export of the community, for example, PV capacities, load patterns, different types of DERs, and charging and discharging patterns of EV and battery storage. It is worth investigating the effects of the factors upon the decentralized trading possibilities.
(2) The decentralized trading mechanisms, Auction Mechanism (AM), Bill Sharing (BS), and Traditional Mechanism (TM) enable participants to trade electricity with more benefits to both demand and supply sides, but the clearing price calculations of AM and BS give the importance to the supply side. The AM computes the clearing price that is a bid price at the equilibrium point, while the clearing price in BS is the weighted average value of offer prices. These calculations give more advantages to sellers rather than buyers.
(3) This business model allows participants to submit their bids and offers manually, so it is not convenient in reality. The next study should consider an agent device that represents a trader with an order generation algorithm. The algorithm could generate energy orders from historical data, energy forecasting, and user preferences so that the energy order will get more effective in the bidding process.
(4) In a decentralized trading model, consumers and prosumers can trade electricity to each other in their community before trading the excess energy to the main grid. The result shows an impact on electricity authorities as they could lose income when their customers participate in the decentralized trading platform rather than the centralized model. Thus, the subsequent study must consider whether the electricity authorities can join the decentralized system as a service provider instead of a central authority.

## Figures and Tables

**Table 1:** Model assessment at community level and individual level.

Models	Self-Consumption	Self-Sufficiency	Cost of Community (THB)	Average Cost of Individual Users (THB)		Participation Willingness Index
				25 Prosumers	75 Consumers	
Centralized Trading Model	0.318	0.248	336,490.914	−5495.441	6509.367	0
Decentralized Trading Model	0.990	0.771	146,558.803	−11,017.153	5626.512	1.000

## Data Availability

https://docs.google.com/spreadsheets/d/1-1qOoClgJ2pzfcnCobB3tiEaH1WqRo5w/edit?usp=sharing&ouid=107495970593559891831&rtpof=true&sd=true.

## Appendix A

1. Self-Consumption

First of all, PV generation ($Q_{PV}^{i,d}$; kWh) for each prosumer (i $\in \;\left\{1,2,3,\ldots ,n_{p}\right\}$) during the particular time $t_{2}-t_{3}$ each day d can be calculated by Equation (A1).

(A1) $$Q_{PV}^{i,d}=Q_{PV}^{i,t_{3}}-Q_{PV}^{i,t_{2}}$$

where $Q_{PV}^{i,t_{2}}$ and $Q_{PV}^{i,t_{3}}$ are the accumulated amount of PV generation at the specified time $t_{2}$ and $t_{3}$, respectively.

With the centralized trading model, the self-consumption ($SC_{Cen}$) is calculated by the ratio between the summation of individual self-consumption consumed by prosumers ($n_{p}$) and the overall PV generation produced by the community during the particular time considered in this paper is $t_{2}-t_{3}$ which can show the differentiation for both models. The individual self-consumption is computed by the individual PV generation $(Q_{PV}^{i,d}$) minus the individual energy export to the main grid under centralized model participation ($Q_{ex,Cen}^{i,\;d})$.

(A2) $$SC_{Cen}=\frac{\sum_{d=1}^{365}\sum_{i=1}^{n_{p}}(Q_{PV}^{i,d}-Q_{ex,Cen}^{i,\;d})}{\sum_{d=1}^{365}\sum_{i=1}^{n_{p}}Q_{PV}^{i,d}}$$

The same way as the decentralized model, the self-consumption ($SC_{De}$) is calculated by the ratio between the summation of individual self-consumption consumed by the community and the overall PV generation produced by the community during the particular time considered in this paper is $t_{2}-t_{3}$. The individual self-consumption is computed by the individual PV generation $(Q_{PV}^{i,d}$) minus the individual energy export to the main grid under decentralized model participation ($Q_{ex,De}^{i,\;d})$.

(A3) $$SC_{De}=\frac{\sum_{d=1}^{365}\sum_{i=1}^{n_{p}}(Q_{PV}^{i,d}-Q_{ex,De}^{i,\;d})}{\sum_{d=1}^{365}\sum_{i=1}^{n_{p}}Q_{PV}^{i,d}}$$

Both values of $SC_{Cen}$ and $SC_{De}$ rank between 0 and 1. The higher value shows the higher the self-consumption is. In general, most of $Q_{ex,De}^{i,\;d}$ are less than $Q_{ex,Cen}^{i,\;d}$, so the better design of the decentralized model should get the higher $SC_{De}$ than $SC_{Cen}$.

2. Self-Sufficiency

First of all, electric loads ($Q_{PV}^{i,d}$; kWh) for each user (i ∈ {1,2,3,…,n}) during the particular time $t_{2}-t_{3}$ each day d can be calculated by Equation (A4).

(A4) $$Q_{L}^{i,d}=Q_{L}^{i,t_{3}}-Q_{L}^{i,t_{2}}$$

where $Q_{PV}^{i,t_{2}}$ and $Q_{PV}^{i,t_{3}}$ are the accumulated amounts of electric loads at the specified times $t_{2}$ and $t_{3}$, respectively.

With the centralized trading model, the self-sufficiency ($SS_{Cen}$) is calculated by the ratio between the amount of total electric loads owned by prosumers that are supplied by the PV generation produced by themselves and the amount of total electric loads in the community during the particular time considered in this paper is $t_{2}-t_{3}$ which can show the differentiation for both models.

(A5) $$SS_{Cen}=\frac{\sum_{d=1}^{365}\left(\sum_{i=1}^{n}Q_{L}^{i,d}-\sum_{i=1}^{n}Q_{im,Cen}^{i,d}\right)}{\sum_{d=1}^{365}\sum_{i=1}^{n}Q_{L}^{i,d}}$$

The same way as the decentralized model, the self-sufficiency ($SS_{De}$) is calculated by the ratio between the consumption amount of total electric loads in community that are supplied by the PV generation produced by the community and the consumption amount of total electric loads in the community during the particular time considered in this paper is $t_{2}-t_{3}$.

(A6) $$SS_{De}=\frac{\sum_{d=1}^{365}\left(\sum_{i=1}^{n}Q_{L}^{i,d}-\sum_{i=1}^{n}Q_{im,De}^{i,d}\right)}{\sum_{d=1}^{365}\sum_{i=1}^{n}Q_{L}^{i,d}}$$

Both values of $SS_{Cen}$ and $SS_{De}$ rank between 0 and 1. The higher value shows the higher the self-sufficiency is. Under the centralized trading model, only prosumers can consume PV generation produced by themselves, so the consumption amount of total electric loads that are supplied by the overall PV generation produced by the community should be less than the amount of load consumption supplied under the decentralized trading model. Therefore, the better design of the decentralized model should get the higher $SS_{De}$ than $SS_{Cen}$.

3. Cost of community energy

With the centralized model, the cost of the community during a particular time $t_{2}-t_{3}$ in one year can be derived as follows:

(A7) $$Cost_{Cen}=\overset{365}{\underset{d=1}{\sum }}\overset{n_{p}}{\underset{i=1}{\sum }}\left[\left(Q_{im,Cen}^{i,d}\;x\;P_{bg}\right)-\left(Q_{ex,Cen}^{i,d}\times P_{sg}\right)\right]+\overset{365}{\underset{d=1}{\sum }}\overset{n_{c}}{\underset{i=1}{\sum }}\left(Q_{im,Cen}^{i,d}\times P_{bg}\right)$$

According to Equations (A6) and (A7), cost of community under the decentralized model during the particular time $t_{2}-t_{3}$ in one year can be derived as follows:

(A8) $$Cost_{De}=\overset{365}{\underset{d=1}{\sum }}\overset{n}{\underset{i=1}{\sum }}\left[\left(Q_{im,De}^{i,d}\times P_{bg}\right)-\left(Q_{ex,De}^{i,d}\times P_{sg}\right)\right]$$

4. Energy bills of individual users

With the centralized model, the energy bills of individual users in one year can be derived below.

(A9) $$Bill_{Cen}^{i}=\overset{365}{\underset{d=1}{\sum }}\left[\left(Q_{im,Cen}^{i,d}\times P_{bg}\right)-\left(Q_{ex,Cen}^{i,d}\times P_{sg}\right)\right]$$

As mentioned in Equations (4) and (5), the energy bills of individual users under the decentralized model during the whole day in one year can be derived as follows:

(A10) $$Bill_{De}^{i}=\overset{365}{\underset{d=1}{\sum }}\left(Cost^{i,d}-Income^{i,d}\right)$$

5. Participation willingness index

(A11) $$P_{willing}=\frac{N_{lower\;cost}}{N}$$

where $N_{lower\;cost}$ represents the number of users who pay less energy cost under the decentralized model than that under the centralized model, N represents total users who trade electricity under a decentralized model.
